# Sub-1.1 nm ultrathin porous CoP nanosheets with dominant reactive {200} facets: a high mass activity and efficient electrocatalyst for the hydrogen evolution reaction†
†Electronic supplementary information (ESI) available: Fig. S1–S14 and Tables S1–S3. See DOI: 10.1039/c6sc05687c



**DOI:** 10.1039/c6sc05687c

**Published:** 2017-01-25

**Authors:** Chao Zhang, Yi Huang, Yifu Yu, Jingfang Zhang, Sifei Zhuo, Bin Zhang

**Affiliations:** a Department of Chemistry , School of Science , Tianjin Key Laboratory of Molecular Optoelectronic Science , Tianjin University and Collaborative Innovation Center of Chemical Science and Engineering (Tianjin) , Tianjin 300072 , China . Email: bzhang@tju.edu.cn

## Abstract

The exploration of a facile strategy to synthesize porous ultrathin nanosheets of non-layered materials, especially with exposed reactive facets, as highly efficient electrocatalysts for the hydrogen evolution reaction (HER), remains challenging. Herein we demonstrate a chemical transformation strategy to synthesize porous CoP ultrathin nanosheets with sub-1.1 nm thickness and exposed {200} facets *via* phosphidation of Co_3_O_4_ precursors. The resultant samples exhibit outstanding electrochemical HER performance: a low overpotential (only 56 and 131 mV are required for current densities of 10 and 100 mA cm^–2^, respectively), a small Tafel slope of 44 mV per decade, a good stability of over 20 h, and a high mass activity of 151 A g^–1^ at an overpotential of 100 mV. The latter is about 80 times higher than that of CoP nanoparticles. Experimental data and density functional theory calculations reveal that a high proportion of reactive {200} facets, high utilization efficiency of active sites, metallic nature, appropriate structural disorder, facile electron/mass transfer and rich active sites benefiting from the unique ultrathin and porous structure are the key factors for the greatly improved activity. Additionally, this facile chemical conversion strategy can be developed to a generalized method for preparing porous ultrathin nanosheets of CoSe_2_ and CoS that cannot be obtained using other methods.

## Abstract 2

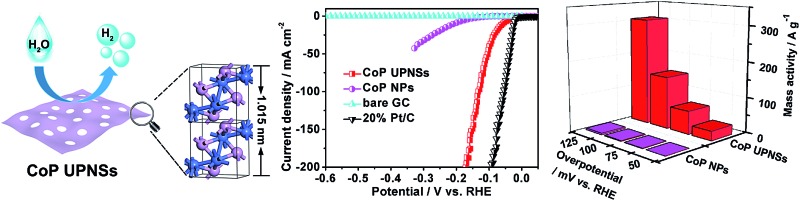
We present a convenient chemical conversion strategy for the synthesis of CoP porous ultrathin nanosheets as highly efficient catalysts for the hydrogen evolution reaction.

## 1. Introduction

Hydrogen produced from water electrolysis is considered to be a promising alternative energy source to fossil fuels by virtue of its environmental benignity and sustainable features.^1–3^ Pt is an excellent electrocatalyst for the hydrogen evolution reaction (HER),^4^ but its practical use is hindered by its high price and rarity. Since the pioneering report of MoS_2_ as an electrocatalyst for HER,^5,6^ low-cost promising candidates composed of earth-abundant elements including metal chalcogenides,^7–10^ carbides,^11–15^ phosphides,^16–22^ phosphosulphides,^23^ and oxides^24,25^ have attracted increasing attention. Although fascinating advances have been made in the search for novel alternatives and the improvement of their HER performances *via* various methods, the materials are mainly restricted to nanoparticles, polycrystalline one-dimensional nanomaterials and thick sheets. Thus, the relatively large sizes and low numbers of active sites of some current catalysts mean that improvement of HER activity, especially the mass activity, is needed. In addition, the catalytic performance of a material is mainly dependent on its exposed crystal facets.^26^ However, the development of a type of electrocatalyst with a large proportion of exposed active crystal planes with high mass activity for HER is still highly desirable.

Two-dimensional (2D) ultrathin nanosheets with thicknesses of several nanometers have been extensively studied as ideal materials for both the fundamental understanding of structure–activity relationships and their promising applications in various fields because of their large specific surface areas, richness of active sites, short electron/carrier transfer distance, structural defects and predominantly exposed crystal facets.^27–36^ For example, greatly enhanced catalytic performances have been achieved by the pioneering 2D ultrathin nanosheets made by the Xie,^27–30^ Wei,^27,28,31^ Yang^32^ and Zhang^33,34^ groups. A huge mass activity for the oxygen evolution reaction has been achieved by well-designed ultrathin CoOOH solid nanosheets.^31^ Thus, some rationally-designed methods including liquid exfoliation,^27–30^ graphene oxide-assisted growth,^34^ topotactic reduction^35^ and conversion^33^ have been successfully developed to produce inorganic ultrathin nanosheets. However, the products are mainly solid, rather than in porous single-crystalline form. In addition, compared to solid materials, porous nanostructures can possess many more active sites and exhibit more facile mass transfer, therefore exhibiting improved chemical and physical properties.^37,38^ More importantly, making ultrathin 2D sheets porous can not only generate more coordinated-unsaturated active atoms, but also allows easy electrolyte infiltration into the inside of the catalysts,^39^ which contributes to providing more active sites to participate in the catalytic reactions and hence ensures energy conversion operating with high-efficiency. However, until now, the development of a facile chemical conversion route to prepare single-crystalline 2D non-layered nanosheets as highly active HER materials, especially endowing them with both ultrathin and porous characteristics, is still a big challenge.

Herein, by choosing CoP, one of the most efficient electrocatalysts for HER and other applications,^40–42^ as the model target, we present a convenient chemical transformation approach to synthesize ultrathin porous CoP nanosheets (CoP UPNSs) with a high proportion of exposed {200} facets, two unit cell-thin thickness and modest distorted atomic structures *via* low-temperature phosphidation of Co_3_O_4_ precursors. Our theoretical calculations and experimental results demonstrate that CoP UPNSs are highly efficient catalysts for HER with a huge mass activity of 151 A g^–1^ at an overpotential of 100 mV. The 2D ultrathin structure with abundant pores and active sites, high fraction of exposed active facets, modest structural disorder of CoP NSs and facile ion/electron transfer are the key factors for the superior catalytic performance. Furthermore, this facile chemical conversion method can be extended to prepare UPNSs of CoSe_2_ and CoS.

## 2. Experimental

### 2.1 Material synthesis

#### 2.1.1 Preparation of ultrathin porous Co_3_O_4_ nanosheets

Co_3_O_4_ nanosheets were synthesized according to the reported literature.^43^ Co(acac)_3_ (100 mg) was dispersed into a mixed solution of 20 mL of ethylene glycol and 4 mL of distilled water under vigorous stirring for 12 h in a 50 mL Teflon-lined stainless-steel autoclave. Then the mixture was treated at 190 °C for 48 h and cooled down naturally. The blue products were the CoO nanosheets and were collected by centrifuging the mixture, washed with ethanol and water many times and then dried under vacuum overnight. The as-prepared CoO nanosheets were heated to 400 °C at a rate of 5 °C min^–1^, and kept at 400 °C for 3 h. They were then cooled to room temperature and the obtained powders were the ultrathin porous Co_3_O_4_ nanosheets. After ultrasonic treatment, the powders were dried *via* vacuum freeze-drying for phosphidation.

#### 2.1.2 Synthesis of CoP ultrathin porous nanosheets (CoP UPNSs)

To obtain CoP UPNSs, Co_3_O_4_ (10 mg) and NaH_2_PO_2_·2H_2_O (2 g) were put in two separate quartz boats with NaH_2_PO_2_·2H_2_O at the upstream side of the furnace. Subsequently, the samples were heated to 300 °C for 120 min in a static Ar atmosphere at a rate of 2 °C min^–1^. After cooling to room temperature, the sample was washed with water and ethanol several times and finally dried at 40 °C overnight.

#### 2.1.3 Synthesis of CoP nanoparticles (CoP NPs)

CoP NPs were synthesized according to the reported literature.^44^ 10 mmol of CoCl_2_·6H_2_O and 40 mmol of NaH_2_PO_2_·2H_2_O were mixed together in an agate mortar and ground to a fine mixture. The mixture was transferred to a ceramic boat and heated to 400 °C for 2 h at a heating rate of 2 °C min^–1^ under an Ar atmosphere. Then the sample was naturally cooled to ambient temperature, washed with water and ethanol several times and finally dried at 40 °C overnight.

#### 2.1.4 Synthesis of ultrathin porous CoSe_2_ nanosheets (CoSe_2_ UPNSs)

To obtain CoSe_2_ UPNSs, 5 mg of Co_3_O_4_ was added into 15 mL of ethylene glycol containing Na_2_SeO_3_ (0.625 mmol) under continuous stirring. After 60 min of vigorous agitation, the dispersion was transferred into a 20 mL Teflon-lined autoclave and maintained at 180 °C for 24 h. The samples were collected and washed three times with ethanol and water, respectively, and then dried at 60 °C for 6 h.

#### 2.1.5 Synthesis of ultrathin porous CoS nanosheets (CoS UPNSs)

To obtain CoS UPNSs, 5 mg of Co_3_O_4_ was added into 15 mL of ethylene glycol containing thioacetamide (40 mg) under continuous stirring. After 60 min of vigorous agitation, the dispersion was transferred into a 20 mL Teflon-lined autoclave and maintained at 120 °C for 5 h. The samples were collected and washed three times with ethanol and water, respectively, and then dried at 60 °C for 6 h.

### 2.2 Material characterization

The structures of the samples were determined using a Hitachi S-4800 scanning electron microscope (SEM, 3 kV). Powder X-ray diffraction (XRD) patterns were collected using a Bruker D8 Focus Diffraction System using a Cu Kα source (*λ* = 0.154178 nm). Transmission electron microscopy (TEM), higher-magnification transmission electron microscopy (HRTEM) and elemental distribution mapping images were taken on a JEOL-2100F system equipped with EDAX Genesis XM2. The thickness of the nanosheets was determined using atomic force microscopy (AFM) (Bruker multimode 8). X-ray photoelectron spectroscopy (XPS) measurements were conducted with a PHI-1600 X-ray photoelectron spectrometer equipped with Al Kα radiation. All binding energies were referenced to the C 1s peak at 284.8 eV.

### 2.3 Electrochemical measurements

Electrochemical measurements were performed with a CHI 660D electrochemical workstation (CH Instruments, Austin, TX) and a typical one-component three-electrode cell was used, including a working electrode, a saturated calomel electrode (SCE) as the reference electrode, and a glassy carbon counter electrode in the presence of 0.5 M H_2_SO_4_ as the electrolyte. The reference electrode was calibrated with respect to an *in situ* reverse hydrogen electrode (RHE), by using two platinum wire electrodes as the working and counter electrodes, which yields the relation *E* (V *vs.* RHE) = *E* (V *vs.* SCE) + 0.245 V. A glassy carbon electrode decorated with catalyst samples was used as the working electrode. In a typical procedure for the fabrication of the working electrode, 4 mg of CoP catalyst and 20 μL of 5% Nafion solution were dispersed in 1 mL of de-ionized water by sonication to generate a homogeneous ink. Then 5 μL of the dispersion (containing 20 μg catalyst) was transferred onto a glassy carbon electrode with a diameter of 3 mm (loading amount: 0.28 mg cm^–2^). The as-prepared catalyst film was dried at room temperature. Polarization data were collected at a sweep rate of 2 mV s^–1^. Electrochemical impedance spectroscopy (EIS) measurements were carried out in the same configuration at *η* = 56 mV or *j* = 10 mA cm^–2^ from 100 kHz to 0.1 Hz.

#### 2.3.1 Mass activity

The mass activity (A g^–1^) values, as shown in Fig. 2c, of different samples were calculated from the electrocatalyst loading *m* (0.28 mg cm^–2^) and the measured current density *j* (mA cm^–2^) at *η* = 50 mV, 75 mV, 100 mV and 125 mV:
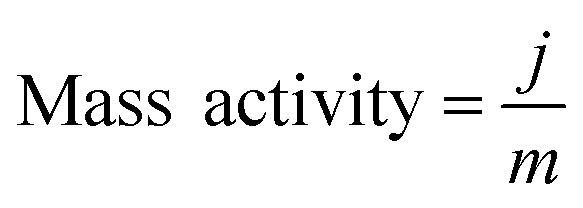


#### 2.3.2 Double-layer capacitance values

Electrochemical capacitance measurements were used to demonstrate the active surface area of the material. The potential was swept between 0.05 to 0.15 V *vs.* RHE five times at each of the given scan rates (10, 20, 40, 60, 80, 100, 120, 160, 200, 250 and 300 mV s^–1^) to obtain the electrochemical capacitance. The cyclic voltammograms for the CoP UPNSs and CoP NPs can be seen in Fig. 3a and b. We chose the capacitive currents at 0.10 V *vs.* RHE, where faradic processes could not be observed in the potential range. The obtained capacitive currents are plotted as a function of scan rate in Fig. 3c and a linear fit measured the specific capacitance to be 7.87 mF cm^–2^ for the CoP UPNSs and 0.358 mF cm^–2^ for the CoP NPs. The specific capacitance for a flat surface is generally found to be in the range of 20–60 μF cm^–2^. We used a value of 40 μF cm^–2^ in the following calculations of the electrochemical active surface area.^45^ 
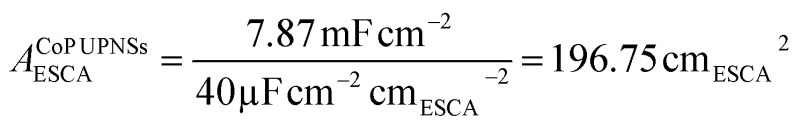
 
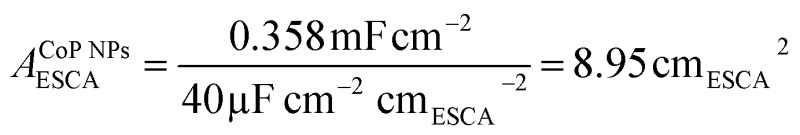


### 2.4 Theoretical calculations

Density functional theory (DFT) calculations were computed by the Vienna Ab initio Simulation Package (VASP). In the DFT calculations, the (100) surface was obtained by cutting bulk CoP along the [100] direction. The thickness of the surface slab was chosen to be that of a two-layer slab of the CoP unit. A vacuum layer as large as 12 Å was used along the *c* direction normal to the surface to avoid periodic interactions. A (2 × 2) supercell was used. The Gibbs free-energy change (Δ*G*_ads_) of H on CoP (100) is defined as follows:Δ*G*_ads_ = Δ*E*_ads_ + Δ*E*_ZPE_ – *T*Δ*S*where Δ*E*_ads_ is the adsorption energy of the atomic H on the CoP (100) surface, Δ*E*_ZPE_ is the difference in zero-point energy (ZPE) between the adsorbed hydrogen and hydrogen in the gas phase, and Δ*S* is the entropy change of one H atom from the absorbed state to the gas phase. Since the H atom is binding on the surface, the entropy of the adsorbed hydrogen can be assumed to be negligible. Therefore, Δ*S* can be estimated by –1/2 × *S*_0_, in which *S*_0_ is the standard entropy of H_2_ in the gas phase at a pressure of 1 bar and pH = 0 at 300 K. In summary, the Gibbs free-energy change (Δ*G*_ads_) of H can be described asΔ*G*_ads_ = Δ*E*_ads_ + 0.24 eV

Δ*E*_ads_ is defined as follows:
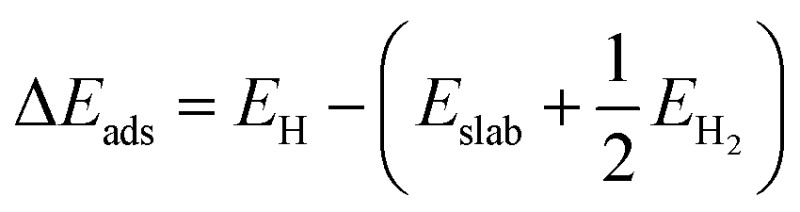
where *E*_H/slab_ is the total energy of the H atom on the CoP (100) surface, *E*_slab_ is the total energy of the CoP (100) surface and *E*_H_ is the energy of the H atom referenced to gas H_2_. The first two terms are calculated with the same parameters. The third term is calculated by setting the isolated H_2_ in a box of 12 Å × 12 Å × 12 Å.

Since there are two surface structures of CoP (100), *i.e.* Co or P terminated, we have therefore calculated the surface energy of both surfaces by the following formula:^46^ 
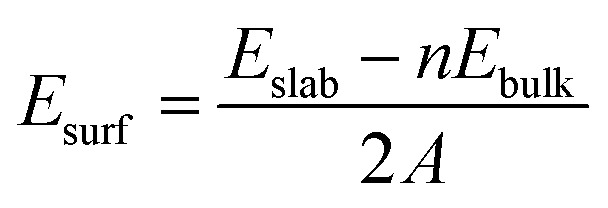
where *E*_slab_ is the total energy of the surface slab, *E*_bulk_ is the total energy of the bulk CoP, *A* is the surface area with a factor of 2 due to each slab containing two surfaces, and *n* is the number of CoP formula units in the slab. A small *E*_surf_ means that the surface is more stable. Thus, the calculated *E*_surf_ values of 1.73 eV Å^–2^ for P terminated (200) and 1.78 eV Å^–2^ for Co terminated (200) indicate that CoP (100) with a P terminated surface is much more stable than that with the Co terminated surface. Thus, we study the P terminated CoP (100) surface in this work.

For the P terminated CoP (100) surface, only P atoms are exposed on the surface. Thus, H atoms will only locate at the top of the P atoms. In our model, there are eight surface P atoms available for H adsorption. The adsorption energy of one H atom (12.5% H coverage, Fig. S11b†) is –0.32 eV, and the adsorption energy will be further reduced to –0.11 eV as the coverage of H is above 75%.

## 3. Results and discussion

**Fig. 1:**
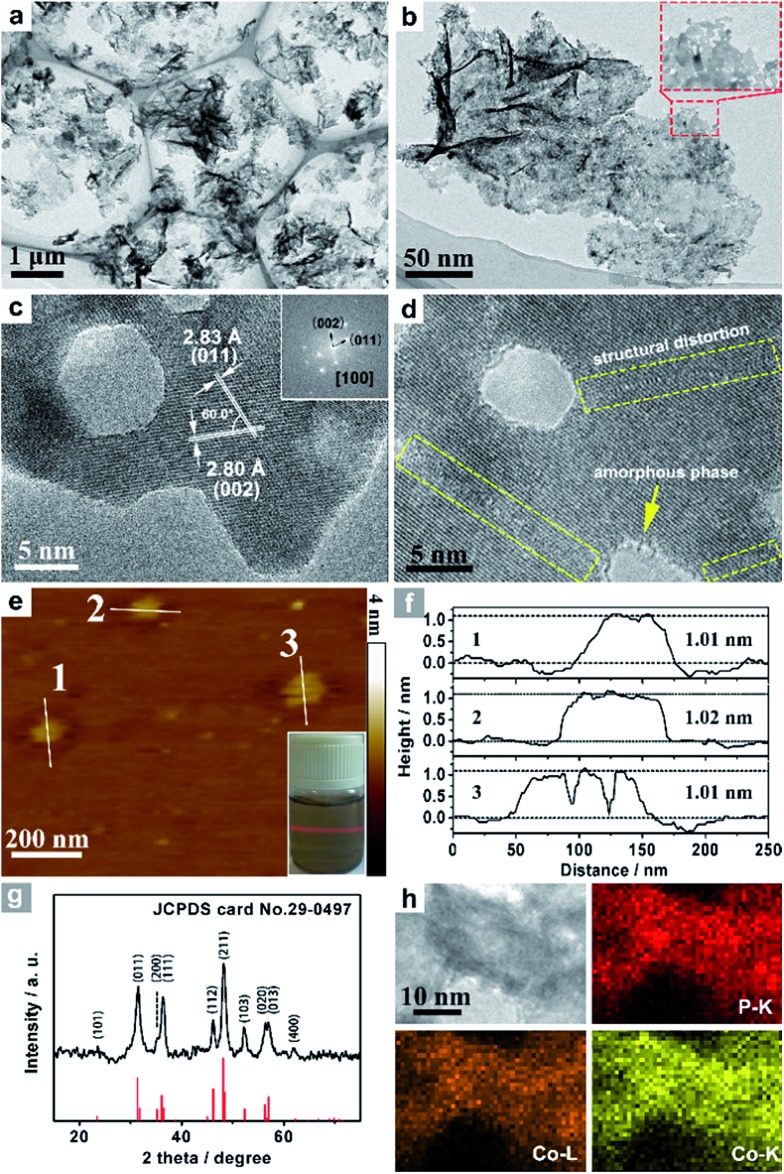
(a, b) TEM images, (c, d) HRTEM images and the associated FFT pattern (inset c) of CoP UPNSs. (e) AFM image and the side-illuminating lighting photo (inset e). (f) The corresponding height profiles of the nanosheets. (g) XRD pattern and (h) STEM-EDS elemental mapping images of CoP UPNSs.

Co_3_O_4_ was selected as the initial material because of its good thermal stability. Firstly, atomically-thick porous Co_3_O_4_ precursor sheets (Fig. S1, ESI†) were synthesized according to a rationally-designed scalable fast-heating strategy developed by the Xie group.^43^ Then the Co_3_O_4_ nanosheets were transformed into CoP through low temperature gas-phase phosphodation using NaH_2_PO_2_ as the phosphorus source. Transmission electron microscopy (TEM) images (Fig. 1a and b) demonstrate that the ultrathin 2D porous structure can be successfully prepared on a large scale, which is of great importance for potential catalytic applications. Moreover, a typical high-resolution TEM (HRTEM) image (Fig. 1c) shows that the nanosheets possess mesopores with diameters of several nanometers. Lattice spacings of 0.280 and 0.283 nm can be attributed to the (002) and (011) crystallographic planes of orthorhombic CoP, respectively (Fig. 1c). A close-up view (Fig. 1d and S2a†) reveals that a mixture of some atomic disorder structures and amorphous areas can be observed clearly. The appearance of structural distortion and the amorphous phase may be associated with strain release due to lattice mismatches of Co_3_O_4_ and CoP.^47,48^ Such structural defects should be conducive to decreasing the surface energy in order to improve the stability of ultrathin 2D sheets.^29^ The associated Fast Fourier Transform (FFT) pattern of the HRTEM image (inset in Fig. 1c) discloses that the porous nanosheet is in single crystalline form with a preferential [100] orientation. The atomic force microscopy (AFM) image and the corresponding height configuration (Fig. 1e and f) indicate that CoP UPNSs possess uniform thickness of about 1.01 nm. This value corresponds to the thickness of two unit cells along the [100] direction of orthorhombic CoP (*a* = 5.076 Å, *b* = 3.279 Å, *c* = 5.599 Å, in JCPDS no. 29-0497), further illustrating that the as-transformed nanosheets possess a preferentially exposed {200} crystal facet with a two unit cell-thin thickness. An obvious Tyndall light-scattering effect is observed by side-illuminating lighting (inset in Fig. 1e), suggesting the formation of a well-dispersed ultrathin 2D sheet colloid. The diffraction peaks in the X-ray diffraction (XRD) pattern (Fig. 1g) can be indexed as orthorhombic CoP (JCPDS no. 29-0497), thus demonstrating the successful conversion from Co_3_O_4_ into CoP. Point-scan energy dispersive X-ray spectroscopy (EDS) analysis (Fig. S3†) and the scanning transmission electron microscopy EDS (STEM-EDS) mapping images (Fig. 1h) indicate the existence and uniform distribution of Co and P. In addition, the specific surface area of UPNSs is 92.23 m^2^ g^–1^ (Fig. S4†). All these results imply that CoP UPNSs with a high proportion of exposed {200} facets and with sub-1.1 nm thickness have been successfully fabricated through the convenient chemical transformation route.

**Fig. 2:**
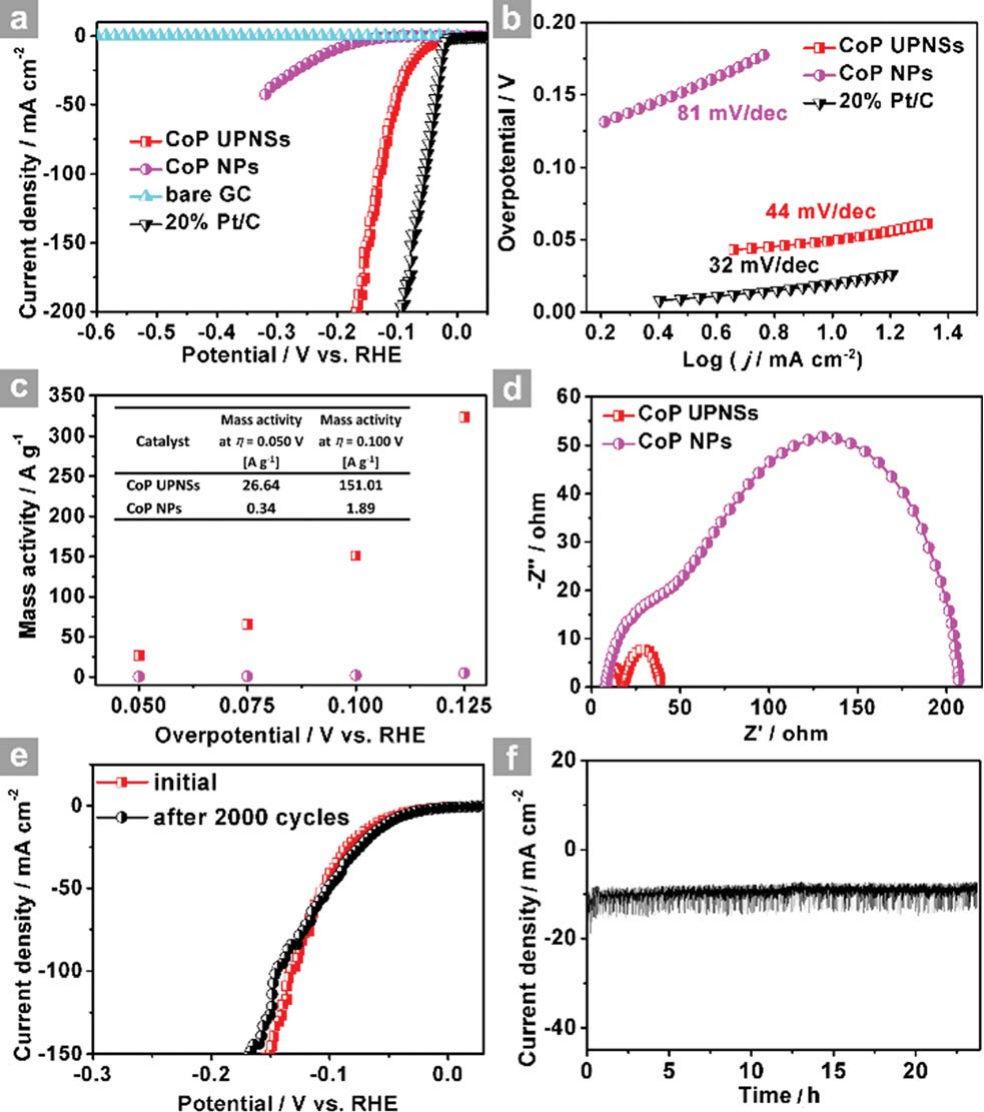
(a) *I*–*R* corrected polarization curves of CoP UPNSs, CoP NPs, bare GC and 20% Pt/C and (b) corresponding Tafel plots of CoP UPNSs, CoP NPs and 20% Pt/C in 0.5 M H_2_SO_4_ at a scan rate of 2 mV s^–1^. (c) Mass activity as a function of the overpotential for CoP UPNSs and NPs. (d) Electrochemical impedance spectra of CoP UPNSs and NPs. (e) Polarization curves of CoP UPNSs initially and after 2000 CV scans. (f) Time-dependent current density curve.

The electrocatalytic HER activity of CoP UPNSs was firstly examined by linear scan voltammetry (LSV) in 0.5 M H_2_-saturated H_2_SO_4_ solution. For comparison, CoP nanoparticles (NPs) (Fig. S5†) and commercial 20 wt% Pt/C deposited on glassy carbon (GC) electrodes with the same amount were also tested under the same conditions. As shown in the *I*–*R* corrected LSV polarization curves (Fig. 2a and S6†), Pt/C unquestionably exhibits the highest performance with negligible overpotential and bare GC is totally inactive towards HER. Surprisingly, for the as-obtained CoP UPNSs, the current densities of 10, and 100 mA cm^–2^ only require overpotentials of 56 mV and 131 mV, respectively, which are much lower than those required for CoP NPs and most reported TMPs under similar conditions (Table S1†). This performance is far superior to most other non-noble metal HER catalysts (Table S2†), indicating the high activity of CoP UPNSs. To probe the HER kinetics, Tafel slopes were calculated. As depicted in Fig. 2b, the Tafel slope for Pt/C is ∼32 mV per decade, which is consistent with the literature values.^1–3,16–21^ The Tafel slope for CoP UPNSs is calculated to be 44 mV per decade, indicating a first-class electrocatalytic activity towards HER with the Volmer–Heyrovsky mechanism.^21^ This value is smaller than that observed for CoP NPs (81 mV per decade) and those of other TMPs (Table S1†). Meanwhile, an extrapolation method applied to the Tafel plot reveals that the exchange current density (*j*_0_) is 0.61 mA cm^–2^, which is the best value for TMP electrocatalysts (Table S1†). Very surprisingly, CoP UPNSs possess a huge mass activity towards HER. For instance, a low overpotential of 100 mV can deliver a mass activity of 151 A g^–1^, which is over 80 times higher than that of CoP NPs (Fig. 2c). All these results demonstrate that CoP UPNSs are highly active for HER with an extremely large mass activity. Electrochemical impedance spectroscopy (EIS) results (Fig. 2d and S7†) demonstrate a smaller interfacial charge-transfer resistance of CoP UPNSs than that of CoP NPs. This greatly accelerating interfacial charge transfer can be ascribed to the improved conductivity^29^ and efficient interfacial contact of 2D porous materials with electrolyte. To evaluate the stability in a strong acid environment, a long-term cycling test was adopted by comparing the polarization curves before and after 2000 CV cycles. The final polarization curve of CoP UPNSs still overlaps with the original one (Fig. 2e). Fig. 2f shows that the catalytic performance remains unchanged for at least 24 h. Additional characterizations clearly show that the original ultrathin porous 2D architecture and composition can be maintained after long-term measurements (Fig. S8 and S9†), revealing that CoP UPNSs are highly stable for HER. Importantly, the CoP UPNSs exhibit almost 100% faradic efficiency for HER (Fig. S10†).

**Fig. 3:**
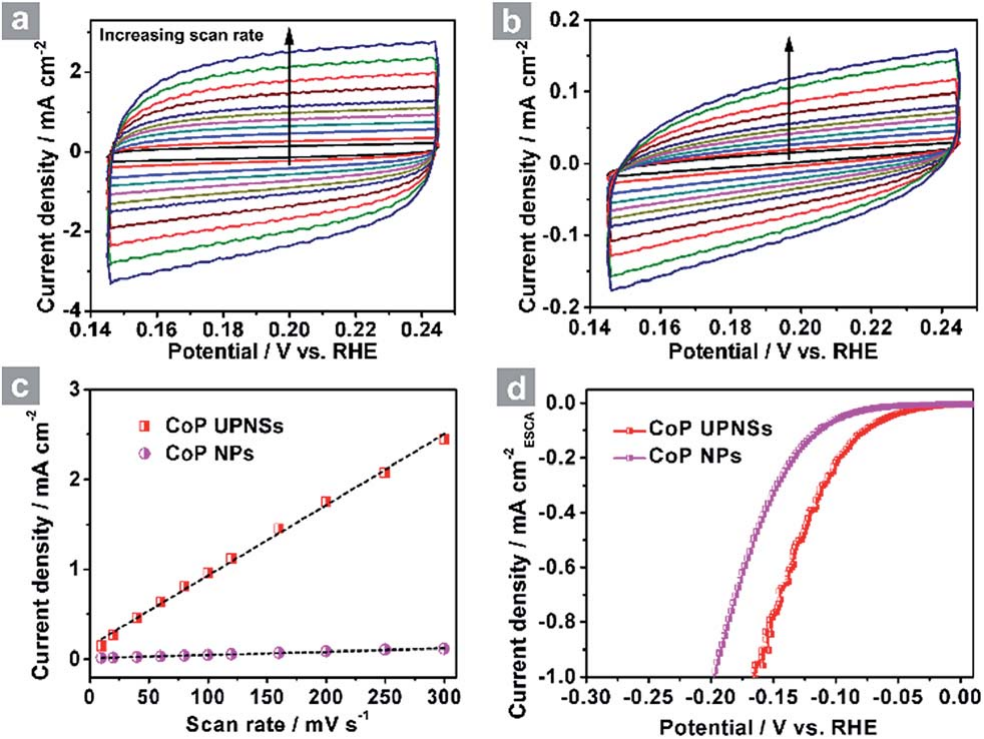
CV curves of CoP UPNSs (a) and CoP NPs (b) with various scan rates, (c) charging current density differences plotted against scan rates. The capacitive currents were measured at 0.10 V *vs.* RHE. (d) The LSV curves from Fig. 2a normalized to the electrochemical active surface area (ECSA).

To elucidate the origins of the outstanding performance of CoP UPNSs, we performed a series of experimental characterizations. The electrochemical active surface areas (ECSA) are usually evaluated by their electrochemical double layer capacitances (*C*_dl_) because of their positive proportion relationship.^49^ As shown in Fig. 3c, CoP UPNSs display a *C*_dl_ value of 7.87 mF cm^–2^, which is 22 times higher than that of CoP NPs (0.358 mF cm^–2^), suggesting a much larger ECSA of CoP UPNSs over the corresponding NPs. Thus, the large ECSA that originates from both the ultrathin and porous characteristics of CoP UPNSs can play an important role on the high activity of the as-converted UPNSs. In addition, the structural distortions and amorphous areas observed in Fig. 1d should make significant contributions to the high activity.^29^ To determine the facet effect and exclude the influence of ECSA on the electrochemical activity of CoP towards HER, the currents were normalized to the relative ECSA. The normalization curves (Fig. 3d) reveal that CoP UPNSs still exhibit a slightly lower onset potential and smaller Tafel slope than CoP NPs. We speculate that the improvement of the normalized activity may be associated with a high proportion of exposed {200} facets in CoP UPNSs.

**Fig. 4:**
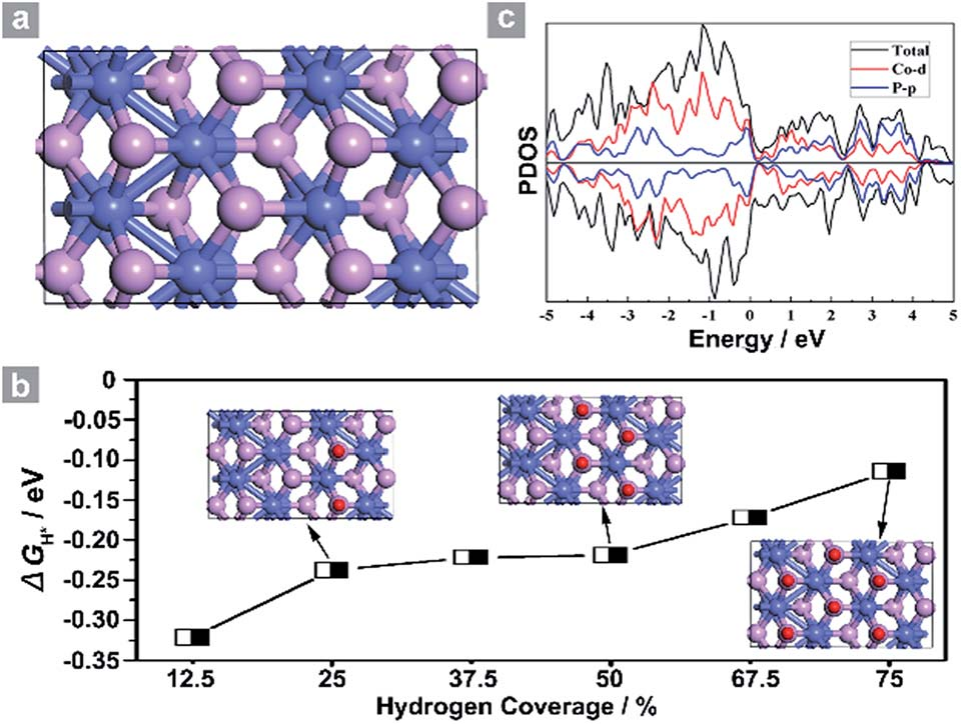
(a) Simulated structure and (b) the dependence of Δ*G*_H*_ on hydrogen coverage *θ*_H*_. (c) Projected density of states of the P terminated CoP (100) facet. Co atoms: blue, P atoms: purple and hydrogen atoms: red.

Next, density functional theory (DFT) calculations were adopted to fundamentally understand the role of the exposed {200} facets. For most electrocatalysts, Δ*G*_H*_ and its coverage dependence are the key descriptors for HER activity.^1–3,45,50–52^ It is believed that the optimal value of |Δ*G*_H*_| is zero.^45,50–52^ For example, the best catalyst, Pt, possesses a Δ*G*_H*_ value of about –0.09 eV.^45,50–52^ Surface formation energy calculations reveal that the stable plane for the (100) facet, one typical plane of {200} facets, is the P terminated CoP (100) surface (Fig. 4a). Fig. 4b shows the dependence of Δ*G*_H*_ on the hydrogen coverage (*θ*_H*_) of the CoP (100) facet. When one hydrogen atom is absorbed on the P terminated surface (12.5% hydrogen coverage), the calculated Δ*G*_H*_ is –0.32 eV (Fig. S11b† and 4b). Although the Δ*G*_H*_ value is comparable with that of most non-precious metal electrocatalysts (Table S3†), the relatively large negative value could not account for such outstanding HER performance.

Interestingly, the calculated Δ*G*_H*_ values shift positively to –0.238 eV and –0.219 eV for the 25% and 50% hydrogen coverages, respectively. The obvious change in the positive direction has been theoretically predicted in some metal phosphides.^45^ Significantly, when *θ*_H*_ is increased to 75%, Δ*G*_H*_ moves positively to –0.114 eV, which is one of the best values for non-precious metal electrocatalysts (Table S3†), suggesting that hydrogen atoms still adsorb strongly on the CoP (100) surface at high *θ*_H*_. For other materials,^45,51^ the high coverage will make Δ*G*_H*_ cross over 0 eV and become a positive value, thus causing the difficulty of hydrogen adsorption. However, for the CoP (100) surface, the near-zero Δ*G*_H*_ at high *θ*_H*_ will lead to the high utilization efficiency of active sites, and thus makes CoP UPNSs with preferentially exposed {200} facets highly active electrocatalysts. In addition, the simulated band structure (Fig. S12†) and the projected density of states (Fig. 4c) reveal the metallic nature of the CoP (100) plane. These metallic characteristics can accelerate electron transfer and thus improve the electrocatalytic performance.

**Fig. 5:**
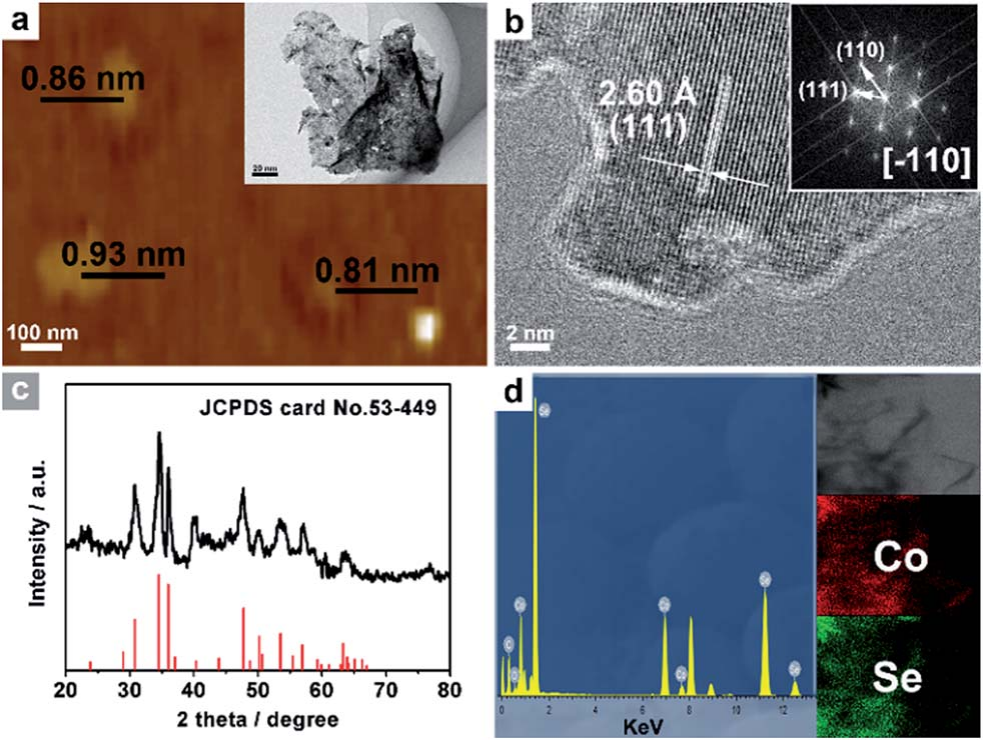
(a) AFM image and TEM image (inset a), and (b) HRTEM images and the associated FFT pattern (inset b) of CoSe_2_ UPNSs. (c) XRD pattern, (d) EDS spectrum and STEM-EDS elemental mapping images (inset d) of CoSe_2_ UPNSs.

This easy chemical transformation strategy can also be utilized to synthesize other UPNSs. For example, the low-temperature selenylation or sulfuration of Co_3_O_4_ precursors can lead to the formation of single-crystalline-like porous ultrathin nanosheets of non-layered CoSe_2_ (Fig. 5 and S13†) and CoS (Fig. S14†), two of the most efficient electrocatalysts for energy conversion,^53–55^ suggesting the generality of our methodology.

## 4. Conclusions

In summary, CoP UPNSs have been successfully synthesized by a handy, robust and efficient chemical transformation strategy of Co_3_O_4_ precursors. This facile strategy endows the as-converted samples with four features: ultrathin (two unit cell-thin thickness), porous, a high proportion of exposed {200} facets, and the coexistence of structural distortion and amorphous areas. The products exhibit outstanding performance for HER: low potential (only 56 and 131 mV are required for current densities of 10 and 100 mA cm^–2^, respectively), small Tafel slope (44 mV per decade), high mass activity (151 A g^–1^ at an overpotential of 100 mV), nearly 100% faradic efficiency and good stability (at least 20 h). The experimental and theoretical results disclose that the structural distortions and amorphous domains, the high amount of active sites, the facile mass/electron transfer caused by both the porous and ultrathin characteristics, the preferentially exposed active facets, the high utilization efficiency of active sites and the metallic nature are key factors for such excellent performance. Importantly, by using this chemical transformation strategy, other metal selenide and sulfide ultrathin porous nanosheets (*e.g.* CoSe_2_, CoS) can be prepared. Our generalized strategy may open a powerful route to synthesize porous ultrathin 2D materials that can't be acquired using the other reported methods. In addition to HER, such novel UPNSs are expected to find other promising energy and catalysis applications (*e.g.* in water oxidation,^28,40^ biomass conversion,^56,57^ and capacitors^58^).
