# Supplementary material for: Sub-1.1 nm ultrathin porous CoP nanosheets with dominant reactive {200} facets: a high mass activity and efficient electrocatalyst for the hydrogen evolution reaction
Source: Chem Sci. 2017 Jan 25;8(4):2769–75. doi: 10.1039/c6sc05687c (PMC5426437; doi:10.1039/c6sc05687c)
Supplement: Supplementary file 1 [file SC-008-C6SC05687C-s001.pdf]

Electronic Supplementary Material (ESI) for Chemical Science.
This journal is © The Royal Society of Chemistry 2017

# Sub-1.1 nm ultrathin porous CoP nanosheets 3 with dominant reactive {200} facets: a high mass activity and efficient electrocatalyst for the hydrogen evolution reaction

Chao Zhang, Yi Huang, Yifu Yu, Jingfang Zhang, Sifei Zhuo, and Bin Zhang*

Department of Chemistry, School of Science, Tianjin Key Laboratory of Molecular Optoelectronic Science, Tianjin University, and Collaborative Innovation Center of Chemical Science and Engineering (Tianjin), Tianjin 300072, China.

Correspondence and requests for materials should be addressed to B.Z. (email: bzhang@tju.edu.cn).

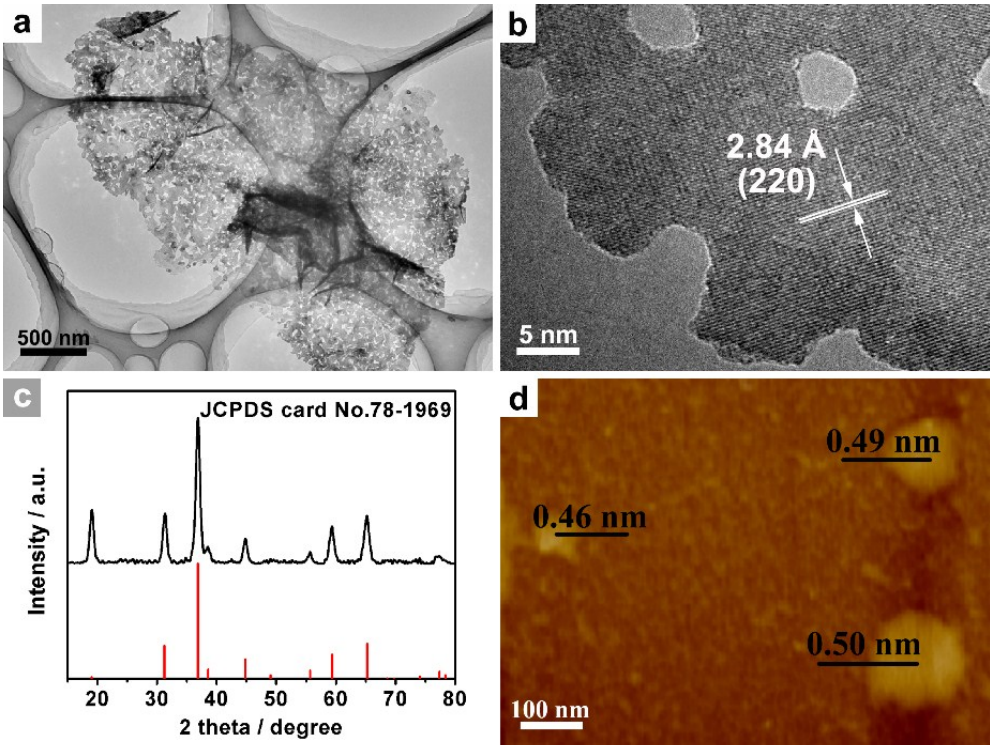


**Figure S1** Characterization of $Co_3O_4$ nanosheets. a,b) TEM and HRTEM image of $Co_3O_4$ nanosheets. c) XRD pattern of $Co_3O_4$ nanosheets. d) AFM image of $Co_3O_4$ nanosheets. It is clearly seen that atomically-thick porous $Co_3O_4$ nanosheets have been successfully synthesized, as shown in Fig. S1a, S1b and S1d. The XRD pattern of $Co_3O_4$ in Fig. S1c illustrates that the product can be indexed to spinel phase $Co_3O_4$ (PDF no. 78-1969).

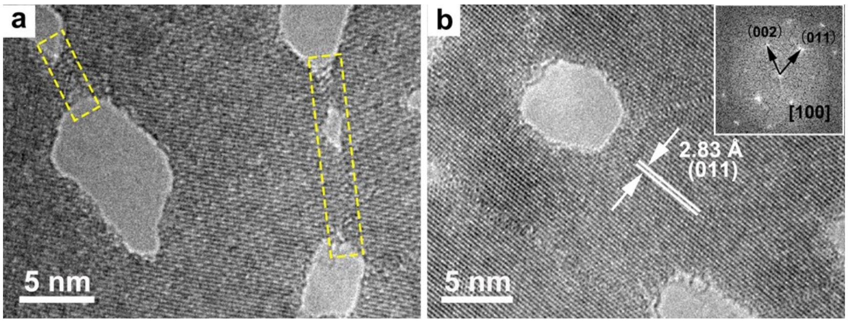


**Figure S2** Additional TEM image to demonstrate more details for CoP UPNSs. a) Additional HRTEM image of CoP UPNSs for demonstrating more details about structural disorder. As shown in Fig. S2a, we could also observe the structural disorders (marked with yellow dotted box) and they all have a certain correlation with pores. b) Additional annotation of lattice spacings and FFT in Fig. 1d to make sure the orientation of CoP UPNSs.

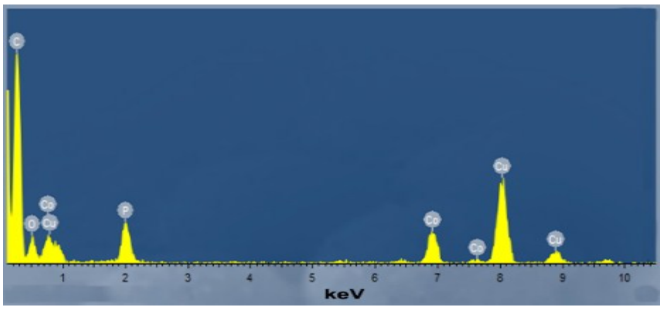


**Figure S3** Elements in CoP UPNSs. The energy-dispersive X-ray spectroscopy (EDS) spectrum of CoP UPNSs. EDS spectrum demonstrates the presence of Co and P in as-prepared CoP UPNSs. The signal of Cu results from the copper substrate.

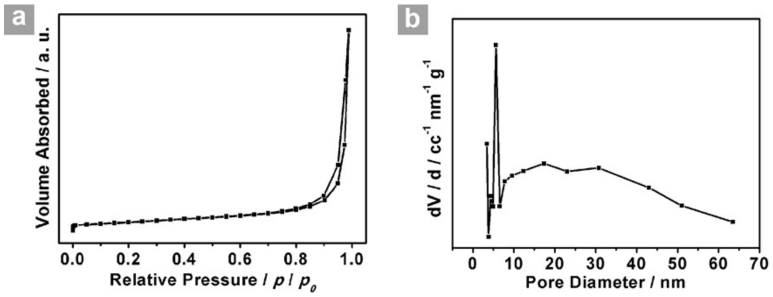


**Figure S4** Nitrogen adsorption/desorption isotherms (a) and pore size distribution (b) of CoP UPNSs. The surface area and pore size distributions of the synthesized materials were determined by nitrogen physisorption using a Quadrasorb SII Quantachrome Instrument. The surface area was calculated using the Brunauer-Emmett-Teller method ($S_{BET}$ = 92.23 $m^2/g$). Pore size distributions were calculated using the Barrett-Joyner-Halenda method from the desorption branch. The presence of mesopores with a size of below 10 nm is attributed to pores in the nanosheets. The pore diameter between 9 and 60 nm can be associated with the stacking pores of nanosheets.

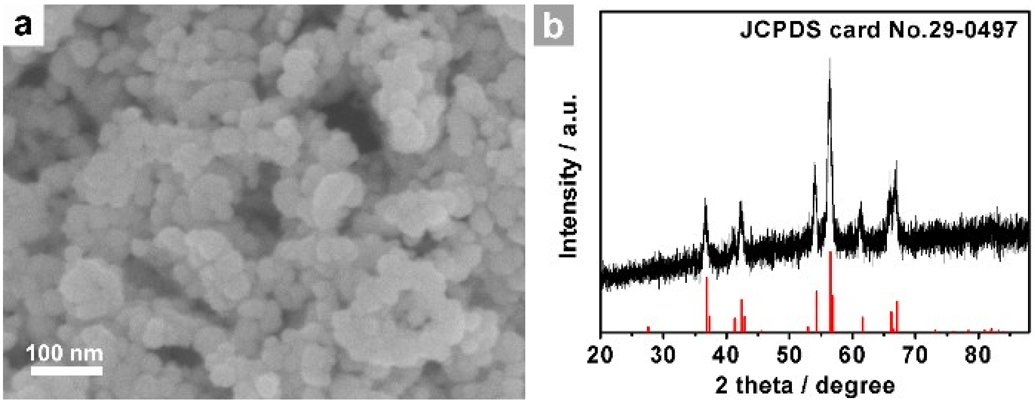


**Figure S5** Characterization of CoP NPs. (a) SEM image and (b) XRD pattern of CoP NPs. The SEM image (Fig. S5a) shows that CoP NPs with a size of about 30 nm have been successfully synthesized. The XRD pattern of the CoP NPs in Fig. S5b shows the CoP NPs have the same spinel phase with CoP UPNSs (PDF no.29-0497).

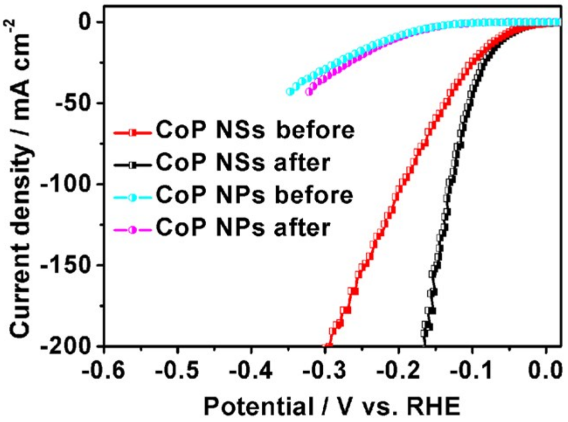


**Figure S6** Linear sweep voltammetry (LSV) curves before and after *I-R* corrected. In order to exactly prove the HER properties of different electrocatalysts, the measured polarization curves should be corrected for all ohmic losses such as wiring, substrates, materials and electrolyte. Data after *I-R* corrections are shown in Fig. 2 in the main text, in which all data have been corrected according to the equation: $E_a = E_b - IR_s$, where $E_a$ is the overpotential after *I-R* correction, $E_b$ is the overpotential before *I-R* correction, $I$ is the corresponding current and $R_s$ is the resistance of the system obtained from electrochemical impedance spectroscopy (EIS, Figure 2d) plots as the first intercept of the main arc (namely the electrode-electrolyte interface).

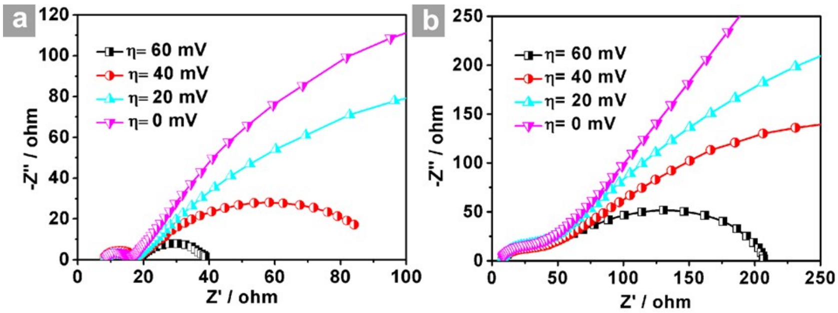


**Figure S7** EIS spectra of CoP nanosheets (a) and CoP nanoparticles (b) at the given overpotentials. The radius of the high-frequency semicircle (small semicircle) for CoP nanosheets is obviously potential-independent, which is associated with the surface porosity response of the CoP nanosheets. The diameter of semicircles at high frequencies can be related to the contact resistance between the catalyst (CoP) and the catalyst support (glassy carbon (GC) electrode). Compared with the diameter of semicircles at high frequencies of CoP nanoparticles, the porous ultrathin CoP nanosheets own a smaller diameter, suggesting the smaller resistance of our sample with the GC substrate. The diameters of the semicircles at low frequencies ($R_{ct}$) are potential-dependent, and the diameters of both samples decrease with increasing applied overpotential, suggesting the faster HER kinetics occurring at higher overpotential. At the same overpotential, the diameter of the semicircles at low frequencies of our ultrathin porous CoP nanosheets is always smaller than the corresponding diameter of CoP nanoparticle, further confirming the faster kinetics for HER.

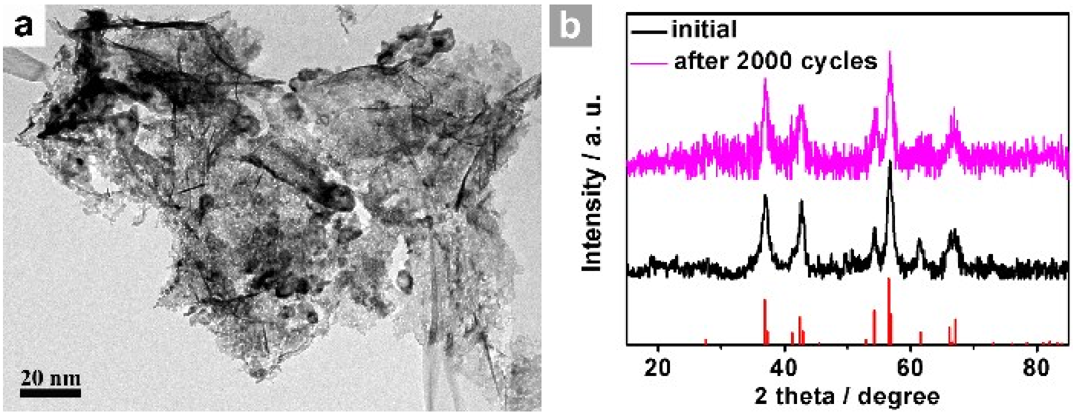


**Figure S8** The stability of CoP UPNSs after a long-term cycling test. a) TEM image of CoP UPNSs after the long-term cycling test and b) XRD pattern of CoP UPNSs before and after the long-term cycling test.

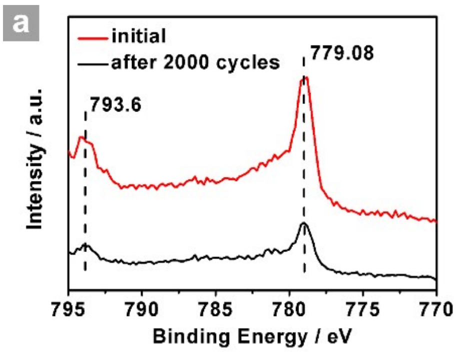


**Figure S9** Comparison of high-resolution XPS spectra of CoP UPNSs before and after a long-term cycling test. High-resolution XPS spectra of a) Co 2p and b) P 2p before and after a long-term cycling test. High-resolution XPS spectra of Co 2p and P 2p regions of CoP UPNSs before and after a long-term cycling test were shown in Fig. S9. The peaks of the initial sample at 779.08 and 129.79 eV are close to the binding energy of Co and P in CoP which are consistent with the reported works.[1] Those results suggest that the CoP have been successfully synthesized from $Co_3O_4$. In addition, we could find that the peaks show no obvious shifts after the stability test which shows that CoP UPNSs are highly stable for HER.

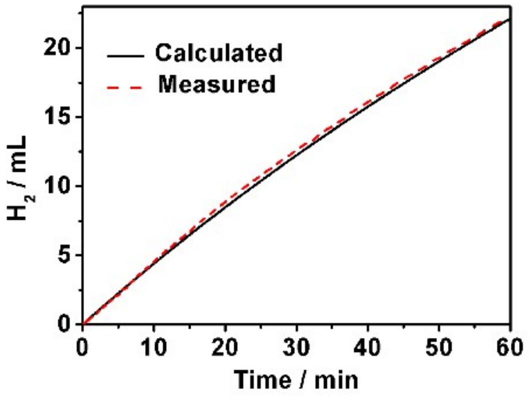


**Figure S10** Faradic efficiency**:** The volume–time curves of CoP UPNSs. The hydrogen evolved from the cathode could be measured by a water-gas displacing method in durability test and data were recorded from the second hour. Q is the cumulative charge (C), F is the Faraday constant (C mol$^{-1}$). The volume of $H_2$ were calculated from the following equation:

$$V_{H_2} mL = \frac{(Q\,C \times 22.4\,L\,mol^{-1} \times 1000)}{F\,C\,mol^{-1} \times 2}$$

As illustrated in Fig. S10, the volume–time curves for the collected $H_2$ match those computed by the cumulative charge volume ratio, indicating that the Faradaic efficiency of the CoP UPNSs was nearly 100%.

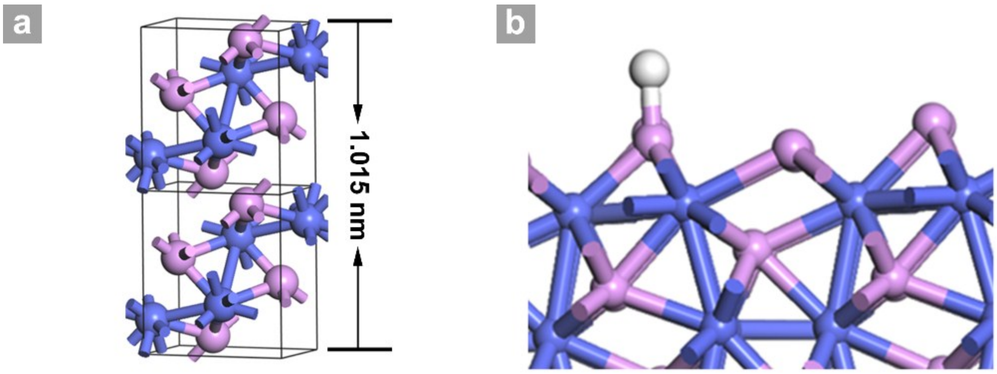


**Figure S11** Simulated structure of a double unit cell (a) and corresponding P terminated CoP (100) surface with one hydrogen atom absorbed (b) in the DFT calculations. Co atoms: blue, P atoms: purple and H atom: white.

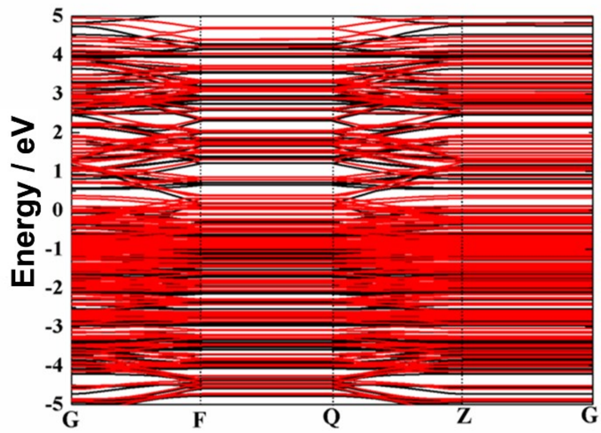


**Figure S12** Simulated band structure of CoP (100) surface. The (100) surface of CoP shows no clear band gap and the electrons near the Fermi level is very active, suggesting it is metallic.

**Table S1. Comparison of HER performance in 0.5 M $H_2SO_4$ for CoP UPNSs with other TMPs catalysts.**

| Catalyst | Overpotential at 10 mA $cm^{-2}$ (mV) | Tafel slope (mV $dec^{-1}$) | Exchange current density (mA $cm^{-2}$) | Ref. |
|---|---|---|---|---|
| CoP | -110 | 54 | 0.16 | 1 |
| $Co_2P$ | -134 | 71 | | 2 |
| FeP | -112 | 58 | 0.22 | 3 |
| $FeP_2$ | | 66 | $1.75\times10^{-3}$ | 4 |
| $Ni_2P$ | -87 | 54 | $7.1\times10^{-2}$ | 5 |
| $Ni_{12}P_5$ | -208 | 75 | $2.857\times10^{-2}$ | 6 |
| MoP | -125 | 54 | | 7 |
| WP | -130 | 69 | 0.29 | 8 |
| $WP_2$ | -148 | 52 | $1.3\times10^{-2}$ | 9 |
| $Co_{1.33}Ni_{0.6}P$ | | 57 | $5.1\times10^{-3}$ | 10 |
| MoP\|S | -64 | 50 | 0.57 | 11 |
| CoP NPs | -203 | 81 | 0.046 | this work |
| CoP UPNSs | -56 | 44 | 0.61 | this work |

Areas are blank if the corresponding data are not mentioned in references.

**Table S2. Comparison of HER performance in 0.5 M $H_2SO_4$ for CoP UPNSs with other non-noble-metal electrocatalysts**

| Catalyst | Overpotential at 10 mA $cm^{-2}$ (mV) | Tafel slope (mV $dec^{-1}$) | Exchange current density (mA $cm^{-2}$) | Ref. |
|---|---|---|---|---|
| $CoSe_2$ NP/CP | -137 | 40 | $(4.9\pm1.4)\times10^{-3}$ | 12 |
| $CoSe_2$ | | 40.8 | $4.1\times10^{-5}$ | 13 |
| $Fe_{0.43}Co_{0.57}S2$ | | 55.9 | $1.6\times10^{-4}$ | 13 |
| $WS_2$ | -142 | 70 | | 14 |
| $MoS_2$ | -187 | 43 | | 15 |
| $CoS_2$ | -145 | 51.4 | $1.88\times10^{-5}$ | 16 |
| $WS_2$ | | 60 | $2\times10^{-5}$ | 17 |
| $CoS_2$/RGO-CNT | -142 | 51 | $6.26\times10^{-2}$ | 18 |
| $Fe_{1-x}Co_xS_2$/CNT | | 46 | | 19 |
| Co-$Mo_2C$ | -140 | 39 | $5.1\times10^{-3}$ | 20 |
| $MoS_2/Ni_3S_2$ | -110 | 83 | | 21 |
| CoP NPs | -203 | 81 | 0.046 | this work |
| CoP UPNSs | -56 | 44 | 0.61 | this work |

Areas are blank if the corresponding data are not mentioned in references.

**Table S3. Hydrogen Adsorption Energy ($\Delta E_{H*}$) and Hydrogen Gibbs Free Energy ($\Delta G_{H*}$) on different non-precious metal electrocatalysts for HER**

| | $\Delta E_{H*}$ (eV) | $\Delta G_{H*}$ (eV) | Ref. |
|---|---|---|---|
| Cu | -0.05 | 0.19 | 22 |
| Ni | -0.51 | -0.27 | 22 |
| W | -0.67 | -0.43 | 22 |
| Mo | -0.61 | -0.37 | 22 |
| Re | -0.56 | -0.32 | 22 |
| Nb | -0.80 | -0.56 | 22 |
| $C_3N_4$@NG | -0.43 | -0.19 | 23 |
| $Mo_2S$ | -0.16 | 0.08 | 24 |
| $WS_2$-1T | 0.01 | 0.25 | 25 |
| $MoSe_2$-1T | 0.42 | 0.66 | 25 |
| $WSe_2$-1T | 0.61 | 0.85 | 25 |
| CoP UPNSs ($\theta_{H*}$=12.5%) | -0.56 | -0.32 | this work |
| CoP UPNSs ($\theta_{H*}$=75%) | -0.354 | -0.114 | this work |

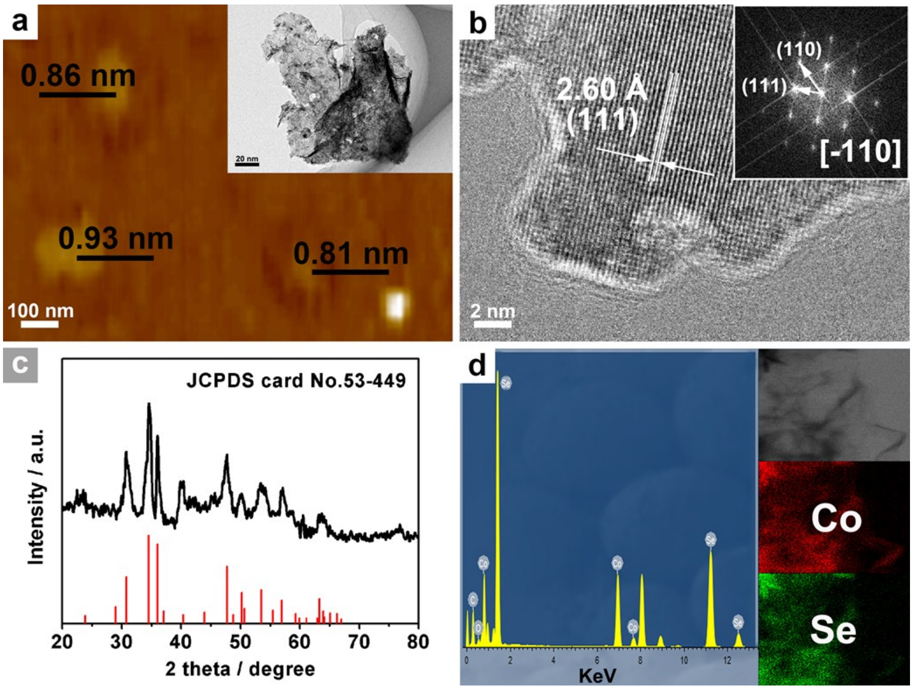


**Figure S13** Characterization of $CoSe_2$ ultrathin porous nanosheets ($CoSe_2$ UPNSs). a) AFM image and TEM image (inset a) and b) HRTEM images and the associated FFT pattern (inset b) of $CoSe_2$ UPNSs. c) XRD pattern, d) EDS spectrum and STEM-EDS elemental mapping (inset d) of $CoSe_2$ UPNSs. The AFM in Figure S13a and the TEM image (inset Figure S13a) suggested that $CoSe_2$ UPNSs were synthesized successfully. Moreover, HRTEM (Figure S13b) and the associated SAED pattern (inset in Figure S13b) showed that the products were single crystalline and the diffraction dots belong to (110) and (111) planes of $CoSe_2$ with a zone axis of [−110]. All the diffraction peaks in the XRD pattern (Figure S13c) can be assigned to orthorhombic $CoSe_2$ structure (PDF no. 53-449) without impurities. Figure S13d showed the EDS and STEM-EDS mapping images (inset in Figure S13d) of as-obtained $CoSe_2$ UPNSs confirming high-purity and uniform distribution of Co and Se elements.

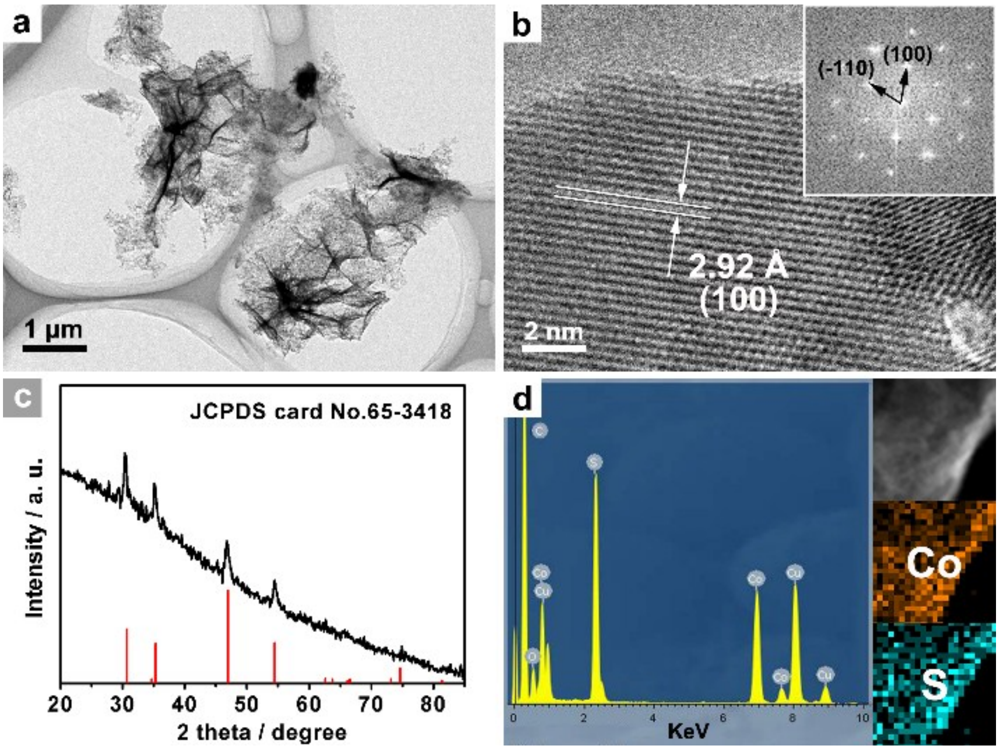


**Figure S14** Synthesis of CoS ultrathin porous nanosheets (CoS UPNSs). a) TEM image, b) HRTEM image and the corresponding FFT pattern (inset b) of CoS UPNSs. c) XRD pattern, d) EDS spectrum and STEM-EDS elemental mapping (inset d) of CoS UPNSs. TEM image (Fig. S14a) showed CoS UPNSs were obtained successfully. Moreover, HRTEM (Fig. S14b) and the associated SAED pattern (inset in Fig. S14b) showed that the products were monocrystalline and the lattice fringe with interplane spacing measured to be 0.292 nm was corresponding to the (100) plane of CoS, respectively. All the diffraction peaks in the XRD pattern (Fig. S14c) can be assigned to hexagonal CoS structure (PDF no. 65-3418) with highly purity. Fig. S14d showed the EDS and STEM EDS mapping images (inset in Fig. S14d) of as-obtained CoS UPNSs confirming the impurity-free and uniform distribution of Co and S elements.

**Reference**


1. P. Jiang, Q. Liu, C. Ge, W. Cui, Z. Pu, A. M. Asiri and X. Sun, *J. Mater. Chem. A*, 2014, **2**, 14634.
2. Z. Huang, Z. Chen, Z. Chen, C. Lv, M. G. Humphrey and C. Zhang, *Nano Energy*, 2014, **9**, 373-382.
3. Z. Zhang, J. Hao, W. Yang, B. Lu and J. Tang, *Nanoscale*, 2015, **7**, 4400-4405.
4. J. Jiang, C. Wang, J. Zhang, W. Wang, X. Zhou, B. Pan, K. Tang, J. Zuo and Q. Yang, *J. Mater. Chem. A*, 2015, **3**, 499-503.
5. Y. Bai, H. Zhang, X. Li, L. Liu, H. Xu, H. Qiu and Y. Wang, *Nanoscale*, 2015, **7**, 1446-1453.
6. Y. Pan, Y. Liu, J. Zhao, K. Yang, J. Liang, D. Liu, W. Hu, D. Liu, Y. Liu and C. Liu, *J. Mater. Chem. A*, 2015, **3**, 1656-1665.
7. Z. Xing, Q. Liu, A. M. Asiri and X. Sun, *Adv. Mater*, 2014, **26**, 5702-5707.
8. Z. Pu, Q. Liu, A. M. Asiri and X. Sun, *ACS Appl. Mater. Interfaces*, 2014, **6**, 21874-21879.
9. H. Du, S. Gu, R. Liu and C. M. Li, *J. Power Sources*, 2015, **278**, 540-545.
10. A. Lu, Y. Chen, H. Li, A. Dowd, M. B. Cortie, Q. Xie, H. Guo, Q. Qi and D.-L. Peng, *Int. J.Hydrogen. Energ*, 2014, **39**, 18919-18928.
11. J. Kibsgaard and T. F. Jaramillo, *Angew. Chem. Int. Ed.* , 2014, **53**, 14433-14437.
12. D. Kong, H. Wang, Z. Lu and Y. Cui, *J. Am. Chem. Soc.*, 2014, **136**, 4897-4900.
13. D. Kong, J. J. Cha, H. Wang, H. R. Lee and Y. Cui, *Energy Environ. Sci.*, 2013, **6**, 3553.
14. M. A. Lukowski, A. S. Daniel, C. R. English, F. Meng, A. Forticaux, R. J. Hamers and S. Jin, *Energy Environ. Sci.*, 2014, **7**, 2608.
15. M. A. Lukowski, A. S. Daniel, F. Meng, A. Forticaux, L. Li and S. Jin, *J. Am. Chem. Soc.*, 2013, **135**, 10274-10277.
16. M. S. Faber, R. Dziedzic, M. A. Lukowski, N. S. Kaiser, Q. Ding and S. Jin, *J. Am. Chem. Soc.*, 2014, **136**, 10053-10061.
17. D. Voiry, H. Yamaguchi, J. Li, R. Silva, D. C. B. Alves, T. Fujita, M. Chen, T. Asefa, V. B. Shenoy, G. Eda and M. Chhowalla, *Nature Mater.*, 2013, **12**, 850-855.
18. S. Peng, L. Li, X. Han, W. Sun, M. Srinivasan, S. G. Mhaisalkar, F. Cheng, Q. Yan, J. Chen and S. Ramakrishna, *Angew. Chem. Int. Ed.* , 2014, **53**, 12594-12599.
19. D. Y. Wang, M. Gong, H. L. Chou, C. J. Pan, H. A. Chen, Y. Wu, M. C. Lin, M. Guan, J. Yang, C. W. Chen, Y. L. Wang, B. J. Hwang, C. C. Chen and H. Dai, *J. Am. Chem. Soc.*, 2015, **137**, 1587-1592.
20. H. Lin, N. Liu, Z. Shi, Y. Guo, Y. Tang and Q. Gao, *Adv. Funct. Mater.*, 2016, **26**, 5590-5598.
21. J. Zhang, T. Wang, D. Pohl, B. Rellinghaus, R. Dong, S. Liu, X. Zhuang and X. Feng, *Angew. Chem. Int. Ed.* , 2016, **55**, 6702-6707.
22. J. K. Nørskov, T. Bligaard, A. Logadottir, J. R. Kitchin, J. G. Chen, S. Pandelov and U. Stimming, *J. Electrochem. Soc.*, 2005, **152**, J23.

23. Y. Zheng, Y. Jiao, Y. Zhu, L. H. Li, Y. Han, Y. Chen, A. Du, M. Jaroniec and S. Z. Qiao, *Nat. Commun.*, 2014, **5**, 3783.
24. T. F. Jaramillo, K. P. Jørgensen, J. Bonde, J. H. Nielsen, S. Horch and I. Chorkendorff, *Science*, 2007, **317**, 100-102.
25. C. Tsai, K. Chan, J. K. Nørskov and F. Abild-Pedersen, *Surf. Sci.*, 2015, **640**, 133-140.
